# Selecting and Evaluating Mobile Health Apps for the Healthy Life Trajectories Initiative: Development of the eHealth Resource Checklist

**DOI:** 10.2196/27533

**Published:** 2021-12-02

**Authors:** Leigh M Vanderloo, Sarah Carsley, Payal Agarwal, Flavia Marini, Cindy-Lee Dennis, Catherine Birken

**Affiliations:** 1 Child Health Evaluative Sciences The Hospital for Sick Children Toronto, ON Canada; 2 Department of Health Promotion, Chronic Disease and Injury Prevention Public Health Ontario Toronto, ON Canada; 3 Institute for Health Systems Solutions and Virtual Care Women’s College Hospital Toronto, ON Canada; 4 Li Ka Shing Knowledge Institute St. Michael’s Hospital Toronto, ON Canada; 5 Lawrence S. Bloomberg Faculty of Nursing University of Toronto Toronto, ON Canada

**Keywords:** eHealth resources, applications, quality assessment, preconception health

## Abstract

**Background:**

The ubiquity of smartphones and mobile devices in the general population presents an unprecedented opportunity for preventative health. Not surprisingly, the use of electronic health (eHealth) resources accessed through mobile devices in clinical trials is becoming more prevalent; the selection, screening, and collation of quality eHealth resources is necessary to clinical trials using these technologies. However, the constant creation and turnover of new eHealth resources can make this task difficult. Although syntheses of eHealth resources are becoming more common, their methodological and reporting quality require improvement so as to be more accessible to nonexperts. Further, there continues to be significant variation in quality criteria employed for assessment, with no clear method for developing the included criteria. There is currently no single existing framework that addresses all six dimensions of mobile health app quality identified in Agarwal et al’s recent scoping review (ie, basic descriptions of the design and usage of the resource; technical features and accessibility; health information quality; usability; evidence of impact; and user engagement and behavior change). In instances where highly systematic tactics are not possible (due to time constraints, cost, or lack of expertise), there may be value in adopting practical and pragmatic approaches to helping researchers and clinicians identify and disseminate e-resources.

**Objective:**

The study aimed to create a set of guidelines (ie, a checklist) to aid the members of the Healthy Life Trajectories Initiative (HeLTI) Canada trial—a preconception randomized controlled clinical trial to prevent child obesity—to assist their efforts in searching, identifying, screening, and including selected eHealth resources for participant use in the study intervention.

**Methods:**

A framework for searching, screening, and selecting eHealth resources was adapted from the PRISMA (Preferred Reporting Items for Systematic reviews and Meta-Analyses) checklist for systematic and scoping reviews to optimize the rigor, clarity, and transparency of the process. Details regarding searching, selecting, extracting, and assessing quality of eHealth resources are described.

**Results:**

This study resulted in the systematic development of a checklist consisting of 12 guiding principles, organized in a chronological versus priority sequence to aid researchers in searching, screening, and assessing the quality of various eHealth resources.

**Conclusions:**

The eHealth Resource Checklist will assist researchers in navigating the eHealth resource space by providing a mechanism to detail their process of developing inclusion criteria, identifying search location, selecting and reviewing evidence, extracting information, evaluating the quality of the evidence, and synthesizing the extracted evidence. The overarching goal of this checklist is to provide researchers or generalists new to the eHealth field with a tool that balances pragmatism with rigor and that helps standardize the process of searching and critiquing digital material—a particularly important aspect given the recent explosion of and reliance on eHealth resources. Moreover, this checklist may be useful to other researchers and practitioners developing similar health interventions.

## Introduction

The ubiquity of smartphones or mobile devices in the general population represents an unprecedented opportunity to reach diverse individuals for preventative health. The use of smartphone apps for the provision of health information, promotion, and intervention has become widespread [1], even sparking a new label, “digitized health promotion” [1]. Electronic health (eHealth) interventions or programs use diverse information and communication technologies (web- or mobile-based) to improve or facilitate health behaviors. Recent systematic reviews of trials evaluating eHealth resources in adolescents and adults observed significant reductions in BMI and improvements in dietary behaviors, physical activity, and self-monitoring [2-4]. In addition, eHealth resources (ie, online web resources or apps) designed to enhance healthy behaviors are appealing: they are highly accessible and sustainable [5], can be tailored to specific populations [6,7], and provide low-cost scalable opportunities for population-wide promotion of health behaviors [8]. Although most interventions using eHealth resources are brief and relatively simple, the content varies greatly and there is a lack of standardized methodology to rigorously evaluate the quality and effectiveness of eHealth resources. However, the proliferation of apps for chronic disease management and prevention poses challenges for clinicians, policy makers, and patients in understanding which apps are most likely to provide benefit.

The prevalence of noncommunicable diseases—including cardiovascular diseases, respiratory diseases, diabetes, and mental health issues—is on the rise worldwide and preventive strategies are urgently needed [9]. To address this issue, the Healthy Life Trajectories Initiative (HeLTI) Canada study was designed. This randomized controlled trial aims to evaluate a preconception to early childhood telephone-based public health intervention with tailored eHealth resources for women and their partners to optimize growth and development among children in Canada [10,11]. This clinical trial uses a Developmental Origins of Health and Disease approach, which is based on the notion that environmental factors interact with genes from preconception to early childhood and that this programming affects a child’s health into adulthood [12]. HeLTI builds upon the diverse clinical trial research capacity in Canada, while harmonizing the intervention and outcome measures with three other international HeLTI trials (in China, India, and South Africa) to generate evidence that will inform national policy and decision-making for the improvement of health and reduction of noncommunicable diseases starting in preconception [13]. The primary objective of HeLTI Canada is to determine whether a 4-phase intervention, from preconception into pregnancy through to infancy and early childhood, can reduce the rates of child overweight and obesity. Secondary objectives aim to reduce child Z-score of BMI (zBMI) and improve zBMI trajectories, cardiometabolic risk factors, health behaviors (nutrition, physical activity, sedentary behavior, and sleep), and development and school readiness at the age of 5 years. Maternal and paternal health outcomes and parenting behaviors are further examined to provide a family-level evaluation.

In the HeLTI Canada trial, participants in the intervention group are assigned to an experienced public health nurse who provides telephone-based collaborative care to support women and their partners to improve their health, modify their health behaviors, or improve their parenting skills. Nurses perform a detailed telephone assessment to identify preconception risk factors or parenting concerns, develop a structured management plan based on family preference, and conduct scheduled follow-up calls to assist the participants in meeting their outlined health goals. Each woman and their partner will be provided with their own secure login to a website that includes personalized web-based eHealth resources based on their specific goals. This selection of eHealth resources will be curated and customized for the participant and will be used by the nurses to provide individual-based care with resources that are convenient and readily accessible to help them achieve their goals. Given the growing popularity of smartphones, tablets, and apps [14], coupled with the noted shift in how individuals consume health information [15], the inclusion of eHealth resources in the HeLTI Canada trial allows the public health nurse and participant to use evidence-based tools to work collaboratively to address identified health needs.

Working groups were created to identify, evaluate, and recommend eHealth resources specific to health goals (or behaviors of focus) that could be used in the HeLTI Canada intervention. These eHealth resources were meant to be easily accessible on a smartphone, tablet, or computer, and provide personalized, innovative, and engaging support to participants with diverse preventive health needs. The selection, screening, and collation of quality eHealth resources was a necessary component to develop and enhance the HeLTI Canada trial intervention. However, the constant creation and turnover of new apps can make this task difficult and time-consuming. Although syntheses of eHealth resources are becoming more common, their methodological and reporting quality require improvement so as to be more accessible to nonexperts [16]. Further, there continues to be significant variation in quality criteria employed for assessment, with no clear method for developing the included criteria. Per the recent scoping review by Agarwal and colleagues [16], there is currently no single existing framework that addresses all six identified dimensions of mHealth app quality (ie, basic descriptions of the design and usage of the resource; technical features and accessibility; health information quality; usability; evidence of impact; and user engagement and behavior change). In instances where highly systematic tactics are not possible (due to time constraints, cost, or lack of expertise), there is still value in adopting practical and pragmatic approaches to helping researchers and clinicians navigate this space. Specifically, guiding principles that researchers and clinicians could use to select quality eHealth resources are an identified need [17,18]. As there were no available guidelines to assist the HeLTI Canada app working groups in this task, we aimed to address this gap. As such, we sought to create a set of guidelines (ie, a checklist) to aid researchers and clinicians in searching, identifying, screening, and selecting eHealth resources for use in research or clinical practice. These guidelines were developed through the experience of HeLTI Canada researchers as they selected eHealth resources for the trial intervention.

## Methods

### Overview

To optimize the rigor, clarity, and transparency of the current guidelines, the PRISMA (Preferred Reporting Items for Systematic reviews and Meta-Analyses) checklists for systematic [19] and scoping [20] reviews were adapted to provide a framework for screening eHealth resources. Reporting guidelines outline a minimum set of items to include in research reports and have been shown to increase methodological transparency, uptake of research findings, and intervention fidelity [21].

### Selection Criteria of eHealth Resources

In this study, eHealth resources were considered for inclusion if the following conditions were met: (1) targeted children or parents influencing behavior change in children; (2) was either a website or app that provided content on health behaviors (physical activity, sedentary behaviors, screen use, nutrition, wellness, healthy weights, active play, healthy habits); (3) had a minimum quality indicator such as a rating of ≥4 stars if the resource was an iOS app or ≥10,000 installs if the resources was an Android app; and (4) was available in English and/or French.

In addition, eHealth resources were excluded for the following reasons: (1) they relied *solely* on data from a paired external device (ie, wearable technology like a Fitbit, with no option of manually inputting data; this was to ensure all apps would be used by all participants without the need to purchase additional hardware), (2) they were not oriented toward individual users (ie, if they were directed toward school or gym programs; this was to ensure open and wide access to the resource), or (3) they had content focused primarily on the management of specific health conditions (ie, heart disease; this exclusion criterion was included to accommodate a universal and more general population approach).

### Information Sources: Locating eHealth Resources

When searching for eHealth resources, multiple information sources were considered, including the following: (1) the Apple Store (iOS) and the Google Play Store (Android); (2) literature reviews of eHealth articles; (3) consultations with eHealth experts (author PA and Practical Apps [working group], Women’s Health College, Toronto, Canada), reputable public organizations and authorities, and government via email; and (4) recommendations from other experts, including family doctors, pediatricians in primary care, and child caregivers.

### Search Strategy

No date restrictions were placed on the search, which was completed in August 2018. Using the previously identified information sources, the following keywords were used to retrieve e-resources: sleep, physical activity, sedentary behaviors, screens, screen time, nutrition, children, smartphone app, online resource, e-resource, eHealth resource, wellness, weight management, healthy weight, play, activity, fitness, development, healthy habits, healthy behavior, behavior change, monitoring, tracking, and health advice. Once retrieved, all resources were exported and saved in an editable Microsoft Excel (Microsoft Corp) spreadsheet via Google Docs (Google LLC) and duplicates were removed manually. Each resource was assigned a unique identification number.

### Process for Selecting Resources

Based on the eligibility criteria, a standardized screening form (Multimedia Appendix 1) was developed; initially, 10 resources were selected to pilot test and refine the form (91.3% agreement across 6 researchers). Next, all selected resources that met the eligibility were reviewed collectively as a team and a final suite of eHealth resources was identified for inclusion. All disagreements in selection were discussed and resolved by consensus and mediated where necessary.

### Methods for Charting and Extracting Data

All eHealth resources were assessed by the 6 reviewers to determine whether they reported on one or multiple health behaviors of interest, and whether the eHealth resource was child- or parent-focused. All data of interest from the eHealth resources (ie, behavior and population of focus, details about the resources) were entered into an Excel sheet stored in Google Docs.

### Quality Assessment of eHealth Resources

With the multitude of health apps identified, it was essential to evaluate the quality of each resource. All eHealth resources were evaluated by a minimum of two team members and—depending on the type of eHealth resource selected—different quality assessment tools were used. When selecting appropriate tools, it is important to consider the needs and preferences of the resource user.

Driven by consultations with eHealth experts, Stoyanov and colleagues’ [22] Mobile App Rating Scale (MARS) was used to evaluate the quality of the apps. The scale contains 23 items, each rated on a 5-point scale (where 1=inadequate, 2=poor, 3=acceptable, 4=good, and 5=excellent) and categorized into three sections: classification, app quality, and satisfaction. The classification section is only used for descriptive purposes. The 19-item app quality section rates apps on 4 subscales: engagement, functionality, aesthetics, and information quality. The subjective quality section contains 4 items evaluating the user’s overall satisfaction. The MARS is scored by calculating the mean scores of the app quality subscales and the total mean score.

For online web resources, the DISCERN tool was used [23]. DISCERN is a brief questionnaire that provides users with a valid and reliable way of assessing the quality of written information on treatment choices for a health problem. The tool consists of 15 key questions plus an overall quality rating. Each question represents a separate quality criterion and is rated on a 5-point scale where 1=no, 2-4=partial, and 5=yes. The rating scale has been designed to help researchers decide whether the quality criterion in question is present or has been “fulfilled” by the eHealth resource.

### Reporting the Individual eHealth Resources and Key Content

**Figure 1:**
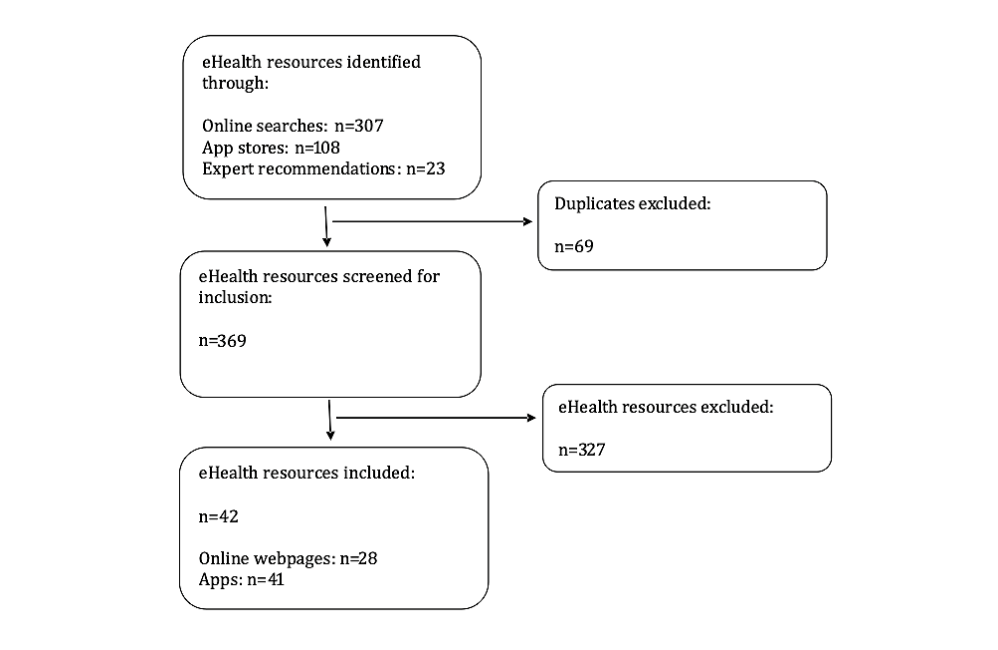
The eHealth resource selection flow diagram.

The total number of resources identified, selected, screened, and assessed for inclusion was recorded (Figure 1). Next, the selected eHealth resources were included in a standardized extraction form and grouped based on health behavior of focus (eg, sleep, physical activity, nutrition, weight management, screen time). Key information about the eHealth resource (type of resource, health behavior, target audience [child or parent], type of content or activities offered, science-backed, etc) was charted.

### Process Refinement and Adoption

Once the initial guidelines were drafted by two members of the working group (LMV and SC), an example online resource and an app were pilot tested by 6 reviewers and 1 mediator to ensure each item or “step” progressed logically and was comprehensive. Following this refinement process, the checklist was modified accordingly. Face validity for the guidelines was achieved by sharing the itemized list with all members of the working group and modifying it further. The final approach was shared and adopted by all members of the HeLTI team to assist with their eHealth resource searching and screening efforts.

## Results

Resulting from the previously described steps, a checklist consisting of 12 guiding principles was systematically developed—organized in a chronological versus priority sequence to aid researchers in searching, screening, and assessing the quality of various eHealth resources in a pragmatic manner (Table 1).

**Table 1:** The eHealth resource checklist.

Section		Item	Checklist item	Present? (✓)
**Objective**				
	Purpose	1	Provides an explicit statement of the objectives being addressed concerning the population and behavior/condition of interest.	
**Methods**				
	Eligibility criteria	2	Specific characteristic of the sources of evidence used as eligibility criteria.	
	Information sources	3	Describes information sources (App Store, online searches, expert consultation). Provides the date the most recent search was conducted.	
	Search	4	Describes the search strategy with enough information so that it is reproducible.	
	Selection of evidence sources	5	States the process for selecting resources.	
	Data mapping and/or charting	6	Describes the methods of charting data.	
	Evaluation and quality assurance	7	Describes the evaluation tools to be used to assess the quality of mHealth (eg, MARS) and eHealth (eg, DISCERN) resources. Note: The needs and preferences of the patient population, as well as the clinical conditions, should be considered when selecting an appropriate evaluation tool.	
**Results**				
	Selection of sources of evidence	8	Provides the number of resources identified, selected, screened, and assessed for inclusion/exclusion.	
	Results of the individual e-resources	9	Presents the relevant data that was charted to help address the study’s objectives, including evidence of effectiveness.	
	Evaluation and quality assurance	10	Uses the MARS (mHealth) or DISCERN (eHealth) tool to assess the quality of the resource.	
**Discussion**				
	Summary of Evidence	11	Summarizes the main findings.	
	Limitations	12	Discusses the limitations of the mHealth/eHealth resource review process.	

## Discussion

### Principal Findings

This paper describes the development of a set of guidelines for pragmatically selecting online resources and apps designed to support a variety of health behaviors as part of the HeLTI Canada trial. Using smartphones for health interventions has the potential to reach many populations, harness the internet’s access to information, and use the latest behavioral science to incorporate nudges and reminders to make positive health decisions the default choice [15,24,25]. However, these novel opportunities for eHealth resources, coupled with their exponential proliferation, are not without their challenges [16]. As expertise in the field of eHealth is not always available to researchers, clinicians, and patients, an evidence-informed checklist to assist with navigating the identification, selection, and assessment of such online web resources and apps is needed. We believe the proposed checklist helps address the gaps outlined in the recent scoping review by Agarwal and colleagues [16], providing a pragmatic approach to evaluating apps by striking a balance between the utilization of standardized quality criteria and the need to conduct expeditious and cost-effective reviews.

### Limitations

During the process of selecting the eHealth resources for the HeLTI Canada trial, it was clear a more rigorous method for searching and selecting mHealth apps was needed. Not surprisingly, practical challenges and limitations were encountered. First, the sheer volume of apps and resources available related to health behaviors (eg, the Apple Store has just over 300,000 apps available [1] and the Google Play Store has approximately 325,000 apps [14]), coupled with the constantly changing content and quick turnover of apps, was a major challenge. Second, because a full download was required to assess the app, evaluators required the necessary hardware on their mobile devices (ie, space and memory) to store the apps. Third, because some of the apps cost money or required in-app purchases, it was at times difficult to fully assess the quality of the app’s contents and features based on the selected quality assessment tools (unless evaluators already had the devices downloaded on their personal devices). Fourth, it was important to ensure that the apps did not endorse private companies and that the recommended apps would not create issues for users’ privacy. Lastly, despite the MARS and DISCERN tools being two of the most widely used eHealth assessment tools, certain dimensions of quality are not captured, such as privacy and security, which may be important to users. Given these limitations, the guidelines and checklist developed to search, screen, and assess eHealth resources should be repeated to confirm their rigor and reliability. With this validation work, we anticipate our checklist and outlined principles will address an important need highlighted by experts to effectively classify and evaluate apps suitable for the most common health conditions through a reliable and valid measurement tool [26].

### Future Considerations and Next Steps

The use of health apps is led by consumers and the self-tracking movement (ie, “the quantified self”). However, the quality of these apps is variable and the evidence to support the effectiveness of these interventions on public and population health is limited or unknown. Other quality assessments focus on understanding the features of apps that may be the catalyst for behavior change [27,28]. Additionally, each health specialty or specific health behavior has developed its own methods to critically appraise eHealth resources [29,30]. Our process provides a more general method to mitigate some of the limitations previously identified in the literature, particularly the large volume of potentially useful apps.

It is contended that many digitized health promotion strategies focus on individual responsibility for health and fail to recognize the social, cultural, and political dimensions of digital technology use. What is particularly noticeable about how digitized health promotion is employed in most programs is that most strategies render health even more individualized and draw attention away from the social determinants of health to a greater degree than ever before. This is despite the current emphasis on health promotion policy that seeks to take a broader approach to alleviate socioeconomic disadvantages and inequities rather than focusing on individuals’ specific health-related behaviors. In the specific context of the HeLTI Canada trial, a public health nurse develops an individualized goal setting plan with each participant; each participant’s context regarding social determinants of health is assessed and the participant is provided with eHealth resources that would work in tandem with their situation, thus helping to alleviate or overcome equity limitations.

### Conclusions

Much like academics have come together to define checklists of critical elements for reporting in clinical trials and systematic reviews, researchers and clinicians planning to use eHealth resources in health behavior interventions require a standardized approach to identify, select, and evaluate these resources. General critical appraisal methods could help researchers from multiple disciplines select and use eHealth tools in their research.

## Appendix

Multimedia Appendix 1 The eHealth resources screening tool.

## Abbreviations

HeLTI: Healthy Life Trajectories Initiative
MARS: Mobile App Rating Scale
PRISMA: Preferred Reporting Items for Systematic reviews and Meta-Analyses
zBMI: Z-score of BMI
