# Supplementary material for: Selecting and Evaluating Mobile Health Apps for the Healthy Life Trajectories Initiative: Development of the eHealth Resource Checklist
Source: JMIR Mhealth Uhealth. 2021 Dec 2;9(12):e27533. doi: 10.2196/27533 (PMC8686460; doi:10.2196/27533)
Supplement: Multimedia Appendix 1 [file mhealth_v9i12e27533_app1.docx]

**Multimedia Appendix 1 – eHealth Resources Screening Tool**

| Screener | Name of eHealth resource | Affiliation (organization, institution) | Year created | App or online? | Behaviour of focus (PA, SB, sleep, nutrition) | Key features | Evidence of effectiveness | Star rating and/or number of installs | Quality assessment score | INCLUDE? |
| --- | --- | --- | --- | --- | --- | --- | --- | --- | --- | --- |
|  |  |  |  |  |  |  |  |  |  |  |
|  |  |  |  |  |  |  |  |  |  |  |
|  |  |  |  |  |  |  |  |  |  |  |
|  |  |  |  |  |  |  |  |  |  |  |
|  |  |  |  |  |  |  |  |  |  |  |
|  |  |  |  |  |  |  |  |  |  |  |
|  |  |  |  |  |  |  |  |  |  |  |
